# *BRCA* Genetic Screening in Middle Eastern and North African: Mutational Spectrum and Founder *BRCA1* Mutation (c.798_799delTT) in North African

**DOI:** 10.1155/2015/194293

**Published:** 2015-02-28

**Authors:** Abdelilah Laraqui, Nancy Uhrhammer, Hicham EL Rhaffouli, Yassine Sekhsokh, Idriss Lahlou-Amine, Tahar Bajjou, Farida Hilali, Jamila El Baghdadi, Abderrahmane Al Bouzidi, Youssef Bakri, Said Amzazi, Yves-Jean Bignon

**Affiliations:** ^1^Laboratoire de Recherche et de Biosécurité-P3, Hôpital Militaire d'Instruction Mohammed V, 10100 Rabat, Morocco; ^2^Laboratoire de Biochimie-Immunologie, Faculté des Sciences, Université Mohammed V-Agdal, 10080 Rabat, Morocco; ^3^Département d'Oncogénétique, Centre Jean Perrin, 58 rue Montalembert, 63011 Clermont-Ferrand, France; ^4^Unité de Génétique, Hôpital Militaire d'Instruction Mohammed V, 10100 Rabat, Morocco; ^5^Laboratoire d'Anatomopathologie, Hôpital Militaire d'Instruction Mohammed V, Equipe de Recherche en Pathologie Tumorale, Faculté de Médecine et de Pharmacie, 10100 Rabat, Morocco

## Abstract

*Background*. The contribution of *BRCA1* mutations to both hereditary and sporadic breast and ovarian cancer (HBOC) has not yet been thoroughly investigated in MENA. *Methods*. To establish the knowledge about *BRCA1* mutations and their correlation with the clinical aspect in diagnosed cases of HBOC in MENA populations. A systematic review of studies examining *BRCA1* in BC women in Cyprus, Jordan, Egypt, Lebanon, Morocco, Algeria, and Tunisia was conducted. *Results*. Thirteen relevant references were identified, including ten studies which performed DNA sequencing of all *BRCA1* exons. For the latter, 31 mutations were detected in 57 of the 547 patients ascertained. Familial history of BC was present in 388 (71%) patients, of whom 50 were mutation carriers. c.798_799delTT was identified in 11 North African families, accounting for 22% of total identified *BRCA1* mutations, suggesting a founder allele. A broad spectrum of other mutations including c.68_69delAG, c.181T>G, c.5095C>T, and c.5266dupC, as well as sequence of unclassified variants and polymorphisms, was also detected. *Conclusion*. The knowledge of genetic structure of *BRCA1* in MENA should contribute to the assessment of the necessity of preventive programs for mutation carriers and clinical management. The high prevalence of BC and the presence of frequent mutations of the *BRCA1* gene emphasize the need for improving screening programs and individual testing/counseling.

## 1. Introduction

Breast cancer (BC) is the most common cancer in women in developed Western countries and is becoming ever more significant in many developing countries [1, 2]. In the Middle East, BC was the leading tumor in women in all cancer registries, accounting for 27.7% to 38.2% of all reported tumors [3]. According to the Middle East Cancer Consortium (MECC), age-standardized incidence rates (ASRs) per 100,000 women were higher among Israeli Jews (93.1), similar to the rates reported in North American and West European countries, and significantly higher than those reported in Cypriot (57.7), Egyptian (49.6), Jordanian (38.0), and Israeli Arab (36.7) women. The lower rates in the other Middle Eastern groups were more similar to rates in Mediterranean Europe, Eastern Europe, and some of Asia and Africa [4]. In Lebanon, the ASR of 71.0 approached Israeli and western values, while the median age at diagnosis for BC was 52 years, compared to 63 years for countries such as the US [5].

Studies of BC in North Africa (including Morocco, Algeria, Tunisia, and Libya) have shown BC patterns similar to those in the Middle East. Over the region, BC accounts for the majority of cancers in women. ASRs range from 23.5 in Algeria and 29 in Tunisia, to nearly 37 in Morocco [6–10], and have overtaken cervical cancer in frequency. As in the Middle East, the average age at diagnosis was more than ten years lower than in the west, at about 48 years in Morocco, Algeria, and Tunisia [8–11], with 7% to 11% of cases younger than 35 years in Morocco and Algeria, respectively. The size and grade of breast tumors are also higher in North Africa than elsewhere [11]. In Libya, the incidence is 18.8 new cases per 100,000 women. Most of the patients present with advanced disease and are often younger than in Europe, in line with the pattern common in North Africa [12].

Although the rapid change in incidence rates of BC in Middle East and North Africa (MENA) countries must be related to environmental lifestyle factors, the young age at onset and high grade suggest the influence of genetic factors such as mutation of* BRCA1*. Indeed, where the overall incidence is low, the contribution of hereditary cases may be relatively high even if the mutation prevalence in the population is comparable.* BRCA1* is responsible for more than 50% of hereditary breast and ovarian cancer (HBOC) families with at least four cancer cases, and of ~15% of families overall [13]. In Western populations, an inherited mutation of this gene confers a lifetime risk of BC of up to 80%, with up to 40% of carriers developing BC by the age of 50 [14]. Penetrance of* BRCA* mutation may be modified both by other risk or protective genes and by environmental factors, most notably reproductive history and diet. The effect of lifestyle on penetrance of* BRCA* mutation is significant, as studies of western populations show that carriers born after 1940 have much higher BC incidence and earlier onset than carriers born before 1940 [15].

The purpose of this paper is to review the current knowledge about* BRCA1* mutations and the correlation between these mutations and the clinical aspect in diagnosed cases of both hereditary and sporadic breast and ovarian cancer in the MECC member countries except for Israel (i.e., Cyprus, Egypt, and Jordan) and Lebanon, as well as in North Africa (Morocco, Algeria, and Tunisia). It seems that no surveys on* BRCA1* mutations have yet been conducted in Libya and no data are available. We chose to exclude Israel from this study because it is a country with a heterogeneous population with diverse geographic and ethnic origins, also with demographics and epidemiology quite different from the rest of the MENA population.

## 2. Methods

We conducted a systematic review of literature on* BRCA1* mutation spectrum and frequencies in MENA cases published on scientific databases up to March 2014. Search terms included breast cancer, heredity breast/ovarian cancer families,* BRCA* genes,* BRCA1* gene, Middle East, and North Africa. Original articles were identified from Cyprus, Egypt, Jordan, Lebanon, Morocco, Algeria, and Tunisia. The included studies had to meet the following criteria: the study must relate to the role of* BRCA1* gene in BC, it should analyze all the coding regions, test specific mutations, or select exons of* BRCA1* gene; the study must provide sufficient information on the deleterious* BRCA1* mutations frequencies. Thirteen relevant references were identified (Table 1), including 10 which used DNA sequencing of all coding axons and flanking intron sequences of the* BRCA1* gene.

Two studies targeted regions containing mutations known to be frequent in other populations: exons 2, 11, and 20 in 135 cases from Jordan [16], axons 2, 8, 13, and 22 in 60 cases from Egypt [17], and one study analyzed 3 specific mutations (c.68_69delAG, c.181T>G, and c.5266dupC) in 30 Egyptian families [18]. These studies used prescreening methods such as single strand conformation polymorphism (SSCP) before sequencing aberrant products or mutation-specific techniques.

Ten studies carried out analysis of the entire* BRCA1* coding region, including 388 familial and 159 young sporadic cases. Several different methods were used, though direct sequencing was the most common. Twenty-six early onset and 40 families with HBOC from Cyprus were sequenced [19, 20]. A sample of 72 cases with a family history of breast or ovarian cancer from Lebanon was screened using direct sequencing [21], as were both sporadic and HBOC families from Morocco [22, 23]. In Algeria, 51 early onset sporadic cases and 11 familial cases were sequenced [24]. A different group used high resolution melting followed by sequencing in 70 BC families from Algeria, followed by multiplex ligation-dependent probe amplification (MLPA) screening for large rearrangements [25]. Finally, 98 HBOC families in Tunisia were screened by direct DNA sequencing [26–28].

For the current paper only deleterious mutations were considered. All deleterious variants are referred to according to the nomenclature used by Human Genome Variation Society (HGVS) (http://www.hgvs.org/) recommendation guidelines, using the A of the ATG translation initiation codon as nucleotide +1. Mutations are also provided using the Breast Cancer Information Core (BIC) (http://research.nhgri.nih.gov/bic/) nomenclature.

## 3. Results

### 3.1. Pathogenic Mutations among Middle Eastern Populations (Table 2)

In Cyprus, two pathogenic* BRCA1* mutations (c.1840A>T and c.5310delG) were identified [19, 20]. Additional variants were detected, including c.4185+3A>G (IVS12+3A>G) which may be pathogenic [20]. Denaturing gradient electrophoresis screening for* BRCA1* gene change showed six germinal mutations among BC Jordanian women [16]. In Lebanon, DNA sequencing revealed five* BRCA1* disease-associated mutations including c.131G>T, c.424C>G, c.536A>G, c.1456T>C, and c.1648A>C [21]. The c.5266dupC mutation in exon 20 was found to be the most frequent* BRCA1* gene mutation among Egyptian women, followed by c.181T>G in axon 5 and c.68_69delAG in axon 2 [17]. The partial mutation screen of exons 2, 13, and 22 identified three distinct mutations in healthy women, including c.68_69delAG, c.4327C>T, and c.5454delC [18].

Mean age at diagnosis was 41 and 42.2 years in Lebanon and Egypt, respectively. The frequency of* BRCA1* mutations ranges from 7.5% in Jordan and Cyprus [16, 19, 20] to nearly 12.6% in Lebanon [21]. Only one patient had a triple negative disease and was not found to have any deleterious mutation in the Lebanese population [21].

### 3.2. Pathogenic Mutations among Northern African Populations (Table 3)

In the Moroccan population, eight distinct* BRCA1* mutations (c.181T>G, c.68_69delAG, c.798_799delTT, c.1016dupA, c.2805delA, c.3279delC, c.5062_5064delGTT, and c.5095C>T) were observed [22, 23]. One mutation, c.4942A>T, was reported in an early onset case without HBOC [23]. Additionally, 32* BRCA1* variants including distinct polymorphisms and unclassified variants were identified [22, 23]. The most frequently detected unclassified variant was c.5117G>C (p.Gly1706Ala) [23]. The* in silico* evidence for c.5117G>C is somewhat stronger, but there is still no cosegregation shown. The c.5117G>C mutation that occurred at highly conserved (GV = 0) was defined as interfering with function (A-GVGD class C55) but has been observed in conjunction with a deleterious mutation in a Tunisian family. However, the cooccurrence of this variant with a deleterious mutation in a Tunisian family suggests that it is neutral [28]. In Algeria, a study [24] revealed five deleterious* BRCA1* mutations among 51 early-onset sporadic cases and four mutations among 11 families (c.46_74del29, c.83_84delTG, c.202+1G>A, and c.798_799delTT). Cherbal et al. [25] reported three distinct pathogenic* BRCA1* mutations (c.83_84delTG, c.181T>G, and c.798_799delTT) and two large genomic rearrangements. Ninety-eight Tunisian familial cases revealed eight mutations, including c.211dupA, c.4041delAG, c.2551delG, c.798_799delTT, c.1504_1508delTTAAA, c.3331_3334delCAAG, c.212+2insG, and c.5266dupC [26–28].

The frequency of* BRCA1* mutations among Moroccan women with HBOC families ranged between 12.8% [22] and 31.6% [23]. The reported frequency in neighbouring countries varied from 8.6% [25] to 36.4% [24] in Algeria and 15.6% [26] to 37.5% [27] in Tunisia. Only 16 families with bilateral BC were accounted for [26–28], in which 5 (31.3%) of them were attributed to pathogenic* BRCA1* mutations. In this subgroup, three cases were carrier of the c.798_799delTT mutation [26, 27] and two cases presented the c.211dupA mutation [28].* BRCA1*-associated breast cancers are more frequently ductal invasive, high-grade carcinomas, less frequently oestrogen and progesterone receptors positive [23, 24, 26]. They are more frequent in triple-negative BC compared to nontriple-negative breast tumors [23, 24, 27].

## 4. Discussion

Mutational analysis in HBOC from Algeria, Morocco, and Tunisia seems to indicate that the North African population has a spectrum of prevalent* BRCA1* mutations, some of which appear as recurrent or founding mutations. The most frequent* BRCA1* gene mutation is c.798_799delTT which was found in roughly all of the North African populations [22–25, 27], suggesting the first non-Jewish founder mutation to be described in this area. Haplotype analysis of some families carrying this mutation revealed the presence of a common allele [24]. None of the founder mutations previously observed among Middle Eastern (Iranian) or Jewish populations were found. To our knowledge, the c.798_799delTT mutation has been identified in Spain [29], in southern Italy [30, 31], and in France [32]. This restricted geographical distribution to close Mediterranean countries could be explained by geographical proximity and migration flow history. c.798_799delTT is frame-shift mutation including two small deletions, that cause truncated protein signal at codon 285.

The remaining mutations occurred at low frequency, and some were previously described as recurrent or founder mutations in other populations. The c.68_69delAG (formerly 185delAG) was identified in Egypt [17, 18]. This mutation is common in Ashkenazi Jews, having attained a 1% carrier frequency, and contributes to 16% to 20% of BC diagnosed before the age of 50 in this population [33]. Markers flanking this mutation have conserved alleles among Ashkenazi carriers, identifying a distinct haplotype [34]. c.68_69delAG was described in all the ethnics including Asian, American, African, and European populations. Since it was replicated in various populations with Arab ethnics including Syria, Iraq, and Yemen, considering this deletion in screening high risk families with Arab ethnics may be helpful [35].

The c.181T>G mutation was observed in Egypt [17], Morocco [22], and Algeria [25]. Haplotype analysis may be useful in establishing whether or not it is the same allele in North Africa. c.181T>G is a founder mutation that can be detected in geographically related populations, notably in Central Europeans [36, 37]. It is interesting that Afro-American populations from the USA have demonstrated that c.181T>G is a recurrent* BRCA1* mutation [37]. Shedding light on the common ancestry of this mutation in two unrelated Afro-American and Eastern European populations may be helpful in finding a logical way to consider it in screening programs of other related populations [35].

The c.5266dupC (5382insC) mutation was described among Egyptian [17] and Tunisian families [26–28]. It is the second most frequently reported mutation in the BIC database. Though being often referred to as a Jewish founder mutation [38], c.5266dupC is common in Central and Eastern Europe with a high frequency among different ethnic populations [39]. It is thought that this mutation probably originated in the Baltic area 38 generations ago, with a gradual decrease in the prevalence going from east to west regions in Europe. A haplotype analysis indicates the likelihood of a single founder both in Europe and in North America for c.5266dupC mutations [40].

The c.5062_5064delGTT mutation was detected in early onset Moroccan BC case with a strong family history of HBOC [22]. It represents the most frequent mutation in Northeast Italy and has been confirmed to be pathogenic using a number of independent approaches [41].

The c.1016dupA mutation was detected in Moroccan families [23]. It has previously been described as one of four founder mutations originating from the Eastern population of Norway [42]. In contrast to other Norwegian founder mutations, the c.1016dupA has also been reported in other ethnic groups. In the BIC database, the c.1016dupA mutation is the 12th most common frame-shift mutation occurring in* BRCA1*. It has been reported that it occurs in populations throughout Europe including Spain, the Netherlands, Austria, and Italy, as well as in Latin America and North America; however, allelotyping results indicated an independent origin of this mutation. The c.1016dupA is a frame-shift mutation due to the insertion of an A at nucleotide acid 1135 of codon 340 in exon 11, which is predicted to lead to a premature stop codon 345 and a truncated protein.

The c.5095C>T mutation was identified in two Moroccan families [23]. It has been described previously in BC families [43–45] and is classified as clinically significant in the BIC database. The amino acid substitution leads to a folding defect in the BRCT domain of the* BRCA1* gene and reduces the proteolytic stability of this domain that interacts with numerous proteins involved in transcription and DNA repair [46]. Functional data and cosegregation suggest that the c.5095C>T mutation is deleterious and predisposes carriers to family history of breast and/or ovarian cancer [46–49].

Interestingly, the c.211dupA mutation was identified among four early onset BC families (27–40 years) originated from the same geographical area in Tunisia [28]. c.211dupA was reported for the first time in two Tunisian families [26] and has never been previously reported in any other population, suggesting that this mutation may therefore be specific for the Tunisian population [28]. The frame shift mutation c.211dupA results in a premature protein termination at codon 79.

Identification of founder and recurrent mutation is an extremely important step towards the improvement of genetic counseling since molecular testing can be targeted to the founder and recurrent mutations allowing for a more rapid and less expensive test [27]. The high frequency of founder mutations, allowing for analyzing a large number of cases, might provide accurate information regarding their penetrance. Furthermore, the evidence of differences in susceptibility and in age onset of cancer among carriers of a specific mutation could make it possible to define the role and importance of risk-modifying factors with the resulting improved disease management [50]. Genetic screening should also be conducted for large genomic rearrangements (LRGs). The identification of LRGs in breast and/or ovarian cancer families has widened the mutational spectrum of the* BRCA1* gene, increasing the number of patients who can benefit from molecular screening [51]. The establishment of the spectrum of* BRCA1* mutations can serve as a cost-effective tool to identify individuals at high risk, such as unaffected carrier relatives who have a defective allele that can be transferred to offspring [52]. This is carried out by either direct sequence or MLPA test as appropriate. Furthermore, germline mutations in other genes, such as* TP53*,* ATM*,* CDH1*,* CHEK2*,* PALB2*,* PTEN*, and* STK11*, should also be studied in North African populations. Estimates of penetrance (8% to 90%) and combined prevalence (5% to 10%) of mutations in these genes in inherited BC are approximate and vary widely, as they are conditional on the study population, mutation, cancer subtype, and syndromic association [53–55].

Published mutation data indicated that UVs remain a problem for genetic counseling. All studies cited one or more variants that could not be classified as either polymorphic/neutral (and of no clinical interest) or deleterious. It has been proposed that the haplotype containing the neutral variants c.536A>G, c.1456T>G, and c.1648A>C together alter the protein function of* BRCA1* and may be pathogenic [56]. Additionally, two variations (c.1456T>C and c.1648A>C), suggested by the authors as pathogenic [21], have since been reported as neutral. This situation complicates genetic counseling and could not contribute in understanding the cause of the high incidence of BC.

The age at which familial cases, regardless of* BRCA1* status, developed BC has been shown to be a decade earlier than western countries. The average age and median age were 48 and 48.5 years old, respectively, in Northern African cases and 65.6% of the patients were younger than 50 years old [57]. This may be explained by the age structure of the population. There are simply far fewer women in their 60s relative to western populations. Thus the later-onset cases are largely missing, leaving possibly the same incidence of young cases, which thus account for a higher proportion. Young age at diagnosis is an indication for referral for* BRCA* testing, and the older age at cancer in* BRCA*-negative families is common.

A majority of tumors were stage II or III and characterized by a larger tumor size probably associated with later diagnosis of Northern African cases. Additionally, frequently positive nodes and negative hormone receptor status were also reported. This excess of high-grade tumors has been described before, with 65% to 86% of tumors being grade II or III in African populations [58–60]. Frequently positive nodes and negative hormone receptor status are both consistent with high-grade tumors. Low- and high-grade breast cancers may represent separate pathways of oncogenesis [61]; thus, the absence of low grade tumors is not explained by delay in diagnosis allowing “progression” to a higher grade. The marked difference in distribution of breast tumor grades between Western and Middle-Eastern/African societies merits further study. One possibility is that low-grade tumors either arise infrequently or do arise but do not develop into palpable tumors initiating medical care. This relative absence of low-grade tumors may contribute to the lower incidence of BC overall in African countries. It may also reflect an ascertainment bias: Western societies have instituted widespread screening programs detecting small low-grade tumors that may go undeclared in developing societies [23]. Two arguments for the biological basis of this difference have been proposed. First, migrants from low-incidence countries gradually take on some of the risk of BC of their host countries, and their descendants have a risk of BC corresponding to the host country [62, 63], arguing for environmental and lifestyle factors in the difference in incidence. On the other hand, studies in the US have shown that BC in Afro-American women is associated with higher grade and poorer prognosis, arguing a biological difference even after socioeconomic differences are controlled and reflecting BC statistics for sub-Saharan Africa [64].

Finally, evaluation of the prevalence of environmental and lifestyle risks is very important in the regional population. These factors are difficult to identify precisely, but their combined effect has serious consequences, as the clear increase in BC incidence in American Ashkenazi* BRCA* carriers born after versus before 1940 shows. Furthermore, other lifestyle factors that may have contributed to BC risk in western populations for multiple generations now but which have only more recently begun to affect the North African population include the use of postmenopausal hormone therapy or oral contraceptives, late age at first birth, increased use of refined foods and chemical food additives, and decreased intake of fresh fruits and vegetables.

## 5. Conclusions

The knowledge of genetic structure of* BRCA1* in North African women will contribute to the assessment of the necessity of the preventive and clinical management of* BRCA1* mutation carriers. Additionally, the high prevalence of BC and the presence of frequent of* BRCA1* mutations emphasize the need for improving screening programs and individual testing/counselling a part of screening policies in North African populations though it will be important to find the appropriate compromise between this strategy and the economic constraints inherent to these countries.

## Figures and Tables

**Table 1:** Details of studies examining BRCA1 in MENA.

Study	Case selection	Region covered	Detection method
Hadjisavvas et al. [19] (Cyprus)	40 familial cases	All	Sequencing

Loizidou et al. [20] (Cyprus)	26 familial cases	All	Sequencing

Atoum and Al-Kayed [16] (Jordan)	135 BC females	Exons 2, 11, and 20	DGGE

Jalkh et al. [21] (Lebanon)	72 familial cases	All	Sequencing

Ibrahim et al. [17] (Egypt)	20 healthy females and 30 familial cases	c.68_69delAG/exon 2 c.181T>G/exon5 c.5266dupC/exon 20	MS-PCR RFLP

El-Debaky et al. [18] (Egypt)	60 familial cases	Exons 2, 8, 13, and 22	SSCP Heteroduplex analysis Sequencing

Tazzite et al. [22] (Morocco)	34 familial cases 6 single cases (<40 years)	All	Sequencing

Laraqui et al. [23] (Morocco)	19 familial cases 102 early-onset sporadic (<45 years)	All	Sequencing

Uhrhammer et al. [24] (Algeria)	13 familial cases 51 young sporadic cases (<38 years)	All	Sequencing MLPA

Cherbal et al. [25] (Algeria)	86 familial cases	All	HRM Sequencing MLPA

Troudi et al. [26] (Tunisia)	34 familial cases	All	Sequencing

Mahfoudh et al. [27] (Tunisia)	16 familial cases	All	Sequencing

Riahi et al. [28]	48 familial cases	All	Sequencing

DGGE: denaturing gradient electrophoresis; MS-PCR: mutagenically separated PCR, SSCP: single-strand conformation polymorphism, RFLP: restriction fragment length polymorphism, HRM: high-resolution melting, MLPA: multiplex ligation-dependent probe amplification.

**Table 2:** Pathogenic mutations among middle eastern populations.

Genetic variant	Consequence	Age at diagnosis	Familial or sporadic	BC or OC	Reference
Lebanon					
c.131G>T	p.Cys44Phe	ni	Familial	BC	[21]
c.424C>G^*^	p.Pro142Ala	ni	Familial	BC	[21]
c.536A>G	p.Tyr179Cys	ni	Familial	BC	[21]
c.1456T>C	p.Phe486Leu	ni	Familial	BC	[21]
c.1648A>C	p.Asn550His	ni	Familial	BC	[21]

Cyprus					
c.1840A>T	p.Lys614X	ni, 40	Familial	BC	[19, 20]
c.5310delG	p.Phe1772SerfsX21	ni, 33	Familial	BC	[19, 20]

Egypt					
c.68_69delAG	p.Glu23ValfsX17	ni	Familial	BC	[17, 18]
c.181T>G	p.Cys61Gly	ni	Familial	BC	[17]
c.4327C>T	p.Arg1443X	ni	Familial	BC	[18]
c.5266dupC	p.Gln1756ProfsX74	ni	Familial	BC	[17]
c.5335delC	p.Gln1779Asnfs14	ni	Familial	BC	[17, 18]

ni: no information; ^*^mutation is not considered deleterious in international databases.

**Table 3:** Pathogenic mutations among Northern African populations.

Genetic variant	Consequence	Age at diagnosis	Familial or sporadic	BC or OC	Reference
Morocco					
c.181T>G	p.Cys61Gly	34	Familial	BC	[22]
c.798_799delTT	p.Ser267LysfsX19	40, 42, 44	Familial	BC	[22, 23]
c.1016dupA	p.Lys1698X	44, 46	Familial	BC	[23]
c.2805delA	p.S896Vfs104	41	Familial	BC	[22]
c.3279delC	p.Ile1859LysfsX3	32	Familial	BC	[22]
c.4942A>T	p.Lys1648X	45	Familial	BC	[23]
c.5062_5064delGTT	p.Val1688del	25	Familial	BC	[22]
c.5095C>T	p.Arg1699Trp	44, 45	Familial	BC	[23]

Algeria					
c.46_74del29	p.Asn16fs	29	Sporadic	BC	[24]
c.46_74del29	p.Asn16fs	37 + 44	Familial	BC	[24]
c.83_84delTG	p.Leu28Argfs x12	26	Sporadic	BC	[24]
c.83_84delTG	p.Leu28Argfsx12	32	Familial	BC	[25]
c.181T>G	p.Cys61Gly	45	Familial	BC	[25]
c.202+1G>A	Splice donor exon 5	25	Familial	BC	[24]
c.798_799delTT	p.Ser267LysfsX19	30, 32, 33, 43, ni	Familial	BC	[24, 25]
c.1817delC	p.Pro606fs	37	Sporadic	BC	[24]
c.2745dupT	p.Ser915fs	36	Sporadic	BC	[24]
c.3715delT	p.Ser1239fs	36	Sporadic	BC	[24]

Tunisia					
c.211dupA	p.Arg71LysfsX80	54, 47, 38, 40, 40, 27	Familial	BOC, BBC, BC	[26, 28]
c.212+2insG	IVS5+2insG	ni	Familial	BC	[27]
c.798_799delTT	p.Ser267LysfsX19	38, 38, 43	Familial	BC	[27]
c.1504_1508delTTAAA	p.Leu502Alafs	32	Familial	BC	[28]
c.2551delG	p.Glu851Asnfs41	45	Familial	BC	[26]
c.3331_3334delCAAG	3450delCAAG	ni	Familial	BC	[27]
c.4041delAG	p.Gly1348AsnfsX6	65	Familial	BC	[26]
c.5266dupC	p.Gly1348AsnfsX6	50, ni, 34, 56, 47	Familial	BOC, BC	[26–28]

ni: no information.
